# The Relationship Between the Average Decreased Times of Estradiol and Early Miscarriage: An Observational Study

**DOI:** 10.1007/s43032-024-01570-3

**Published:** 2024-05-06

**Authors:** Fangxiang Mu, Chen Wang, Xiaofeng Li, Fang Wang

**Affiliations:** Department of Reproductive Medicine, Lanzhou University Second Hospital, No. 82 Cuiying Men, Chengguan District, Lanzhou, 730030 Gansu China

**Keywords:** The average decreased times of estradiol, Early miscarriage, Estradiol, Threshold effect, Miscarriage

## Abstract

Decreased estradiol (E2) levels are associated with early miscarriage (EM), but the relationship between decreased times of E2 and EM has not been reported. We aimed to investigate the relationship between the average decreased times of E2 (ADTE) and EM. Women with a history of miscarriage were retrospectively recruited from the Reproductive Center of Lanzhou University Second Hospital (Lanzhou, China) between September 2019 and February 2022. Based on pregnancy outcome, they were divided into ongoing pregnancy group (*n* = 359) and EM group (*n* = 104). In addition, subgroup analyses were performed for the number of previous miscarriages and whether E2 levels decreased continuously. The exposure and outcome variables were ADTE and miscarriage before 12 weeks of gestation, respectively. Totally, 1171 patients were recruited and 463 patients were finally analyzed. ADTE was associated with EM (odds ratio [OR] = 1.346, 95% confidence interval [CI]1.154-1.571, *P* < 0.001). When ADTE ≥ 2.5, the EM risk increased 1.17-fold compared to patients with 0-1.249 times (OR = 2.170, 95% CI 1.144-4.117, *P* = 0.018). Moreover, a threshold effect existed in the ADTE and the risk of EM with a value of 4.9 times. When exceeding 4.9 times, the EM risk increased 4.713-fold for each increased unit (OR = 5.713, 95% CI 1.255-23.170, *P* = 0.024). Subgroup analysis showed that ADTE had a greater effect on the occurrence of EM in women with a history of 1-2 miscarriages than in women with 3 miscarriages. Decreased E2 was a risk factor for EM regardless of whether it dropped continuously or not. In conclusion, our study identifies a potential link between ADTE and early miscarriage risk in women with prior miscarriages, yet cautious interpretation is necessary due to inherent design limitations. Further research with prospective designs and large population samples is essential to validate ADTE's utility as a predictive indicator for early miscarriage in clinical settings.

## Introduction

Early miscarriage (EM) is a common event in human pregnancy, accounting for 10% to 20% of clinical pregnancies [1]. Approximately 12% of women experience EM and 1-5% of pregnancies end in recurrent miscarriage [2, 3]. EM, also known as fetal death, pregnancy loss, or spontaneous abortion, is defined as intrauterine pregnancy with an empty gestational sac or a gestational sac containing an embryo or fetus without heart activity before 12 weeks of gestation [3]. Pregnancy condition is usually clinically monitored via transvaginal ultrasound [4]. Moreover, progesterone and human chorionic gonadotropin (hCG) are the most common serum markers to assess pregnancy with an uncertain ultrasound finding, but their sensitivity and specificity are clinically unsatisfactory [5–8]. Numerous studies, however, have found a strong relationship between estradiol (E2) levels and pregnancy outcomes in recent years [9–11].

E2 has stimulating effects on endometrial hyperplasia, myometrial thickening, blood supply, and uterine contractility [12]. Specifically, E2 is critical for the onset and maintenance of pregnancy, as established in existing literature. Early pregnancy E2 concentrations reflect the viability of the dominant follicle and corpus luteum performance. Studies indicate that lower serum E2 levels in women undergoing spontaneous abortions contrast with those in viable pregnancies, underscoring a potential link to follicular and luteal health. Recent research highlights discrepancies in E2 levels between miscarriages and successful pregnancies, pointing to compromised ovarian responsiveness. E2's immunomodulatory capacity in the uterine setting, which may mitigate allogeneic fetal rejection, could elucidate the heightened miscarriage risk associated with E2 inadequacy [13, 14]. However, the results of those studies may not be universal, as they focused on changes in E2 levels at certain points, which may be influenced by the testing standard and time [5, 15–17]. Traditional research methodologies overlook the overall pattern of E2 level changes throughout early pregnancy. Although the average decreased times of E2 (ADTE) does not reflect the dynamics of E2 levels per se, it can reveal how often E2 levels decrease. This metric may significantly contribute to understanding the relationship between changes in E2 levels and the risk of early miscarriage. Notably, frequent declines in E2 levels could signal insufficient hormonal support, thereby elevating miscarriage risk. Therefore, we aimed to investigate the relationship between the E2 concentration across the first trimester (<10 weeks) and EM. ADTE may as a miscarriage warning in early pregnancy is beneficial to women at risk of miscarriage. It may also guide clinical treatment and shared decision-making with patients.

## Materials and Methods

### Study Design and Population

**Fig. 1:**
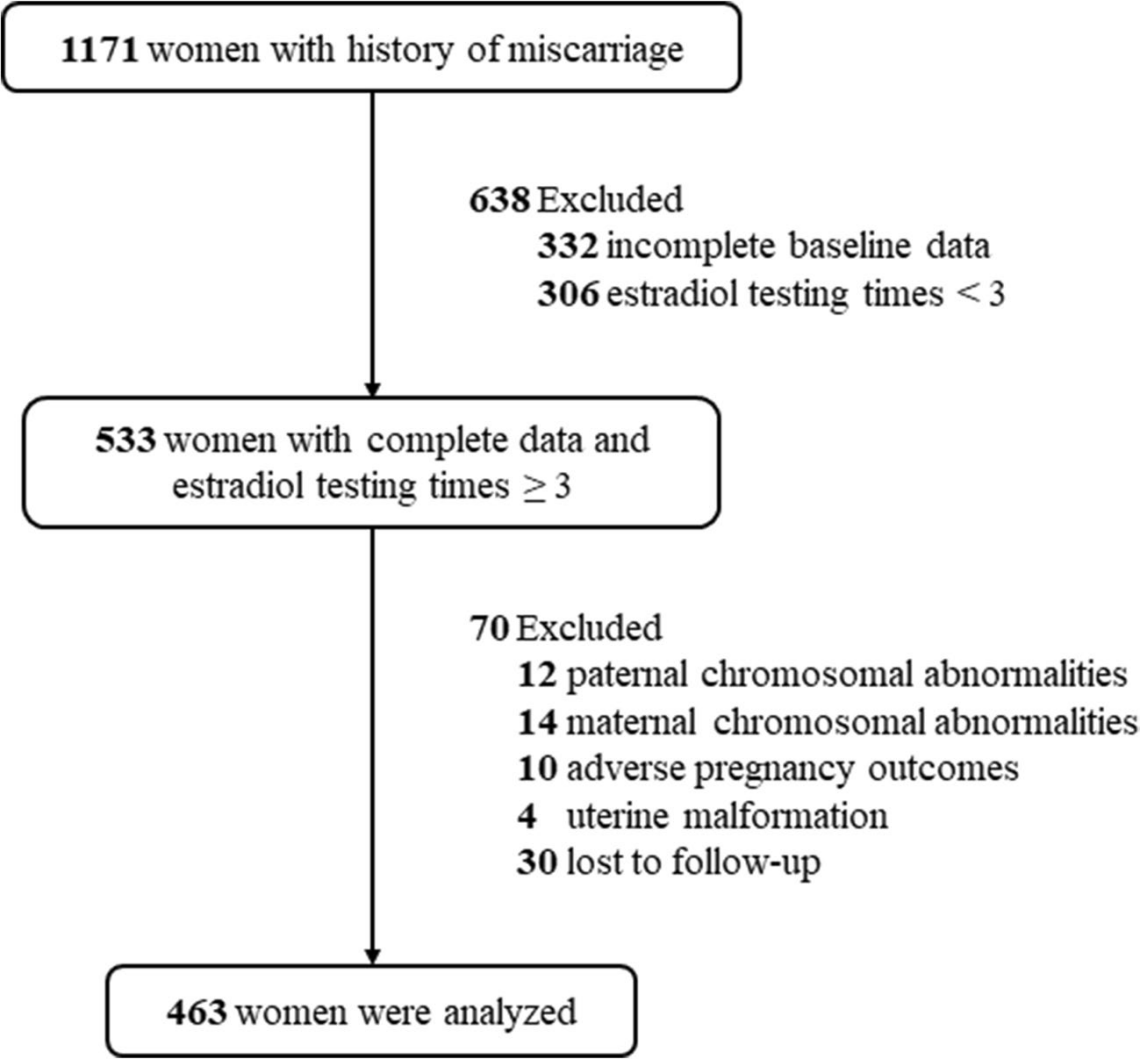
Flow chart for patient selection

This was a retrospective study, recruiting 1171 patients with previous miscarriages who visited the Reproductive Center of Lanzhou University Second Hospital (Lanzhou, China) from September 2019 to February 2022. Finally, 463 women meeting the inclusion criteria were analyzed in this study (Fig. 1). Patients were divided into the early miscarriage group (*n* = 359, cases) and the ongoing pregnancy group (*n* = 104, controls). This study was performed in line with the principles of the Declaration of Helsinki. Approval was granted by the Ethics Committee of Lanzhou University Second Hospital (approval date: 29 Oct. 2019 / approval No. 2019A-231). Informed consent was obtained from the patient.

### Participants

Patients were only included if they (1) were 18-45 years of age, (2) had a history of miscarriage ≥ 1, (3) were naturally conceived, (4) had complete baseline, pregnancy examination, and medication data, and (5) underwent at least three times of serum E2 tests within 12 weeks of gestation.

Exclusion criteria were as follows: abnormal chromosome karyotype in couples and/or embryo chromosomal abnormalities, congenital uterine malformations (septate uterus, unicornuate uterus, bicornuate uterus, and duplex uterus as stated by the American Fertility Society) with no surgical correction performed at this pregnancy, multiple pregnancies, infertility, and adverse pregnancy outcomes (hydratidiform mole, ectopic pregnancy and birth defects, among others). Women who had no childbearing demands and those with missing data were also excluded from the study.

### Data Collection

Demographic characteristics were obtained, consisting of maternal age, body mass index (BMI), age at menarche, menstruation regularity, previous live births, number and type of previous miscarriage, and pre-existing diseases (none, autoimmune disease, endocrinopathy, gynecological disease, others). Basal values of β-human chorionic gonadotropin (β-hCG), E2, and progesterone were provided as references. Additionally, estrogen and progesterone (progesterone injection, progesterone capsules, progesterone gel, and dydrogesterone) use were observed due to the possible interference effects to this study.

### Experimental Indexes and Primary Outcomes

The ADTE was our experimental index. Patients had their E2 levels measured on the second day of the visit, and the next measurement time depending on their condition. Because the number of tests varied among patients, we normalized the data for comparison: ADTE = decrease times / total test times × 10 times. Miscarriage (including biochemical pregnancy, and clinically confirmed miscarriage) occurring before 12 weeks of gestation was the primary outcome of this study. The decrease times refer to the number of drops in the E2 level. Any E2 level lower than the previous measurement was counted as one decrease time.

### Statistical Analysis

Statistical analyses were performed by R studio version 2022.02.1 + 461 and R version 4.1.3 (R Foundation for Statistical Computing Vienna, Austria). Baseline characteristics were described as means ± standard deviations (SD) or median (quartile), or numbers plus percentages. Continuous variables were evaluated using a group t-test or Wilcoxon signed-rank test, while ordinal variables were analyzed by a Wilcoxon signed-rank test or chi-square test. And Chi-square test or Fisher's exact test was adopted for categorical variables analysis. All statistical tests were two-sided tests. *P* < 0.05 was considered statistically significant.

To investigate whether ADTE was associated with early pregnancy outcomes in women with a history of miscarriage, our statistical analysis consisted of four major steps.

**Fig. 2:**
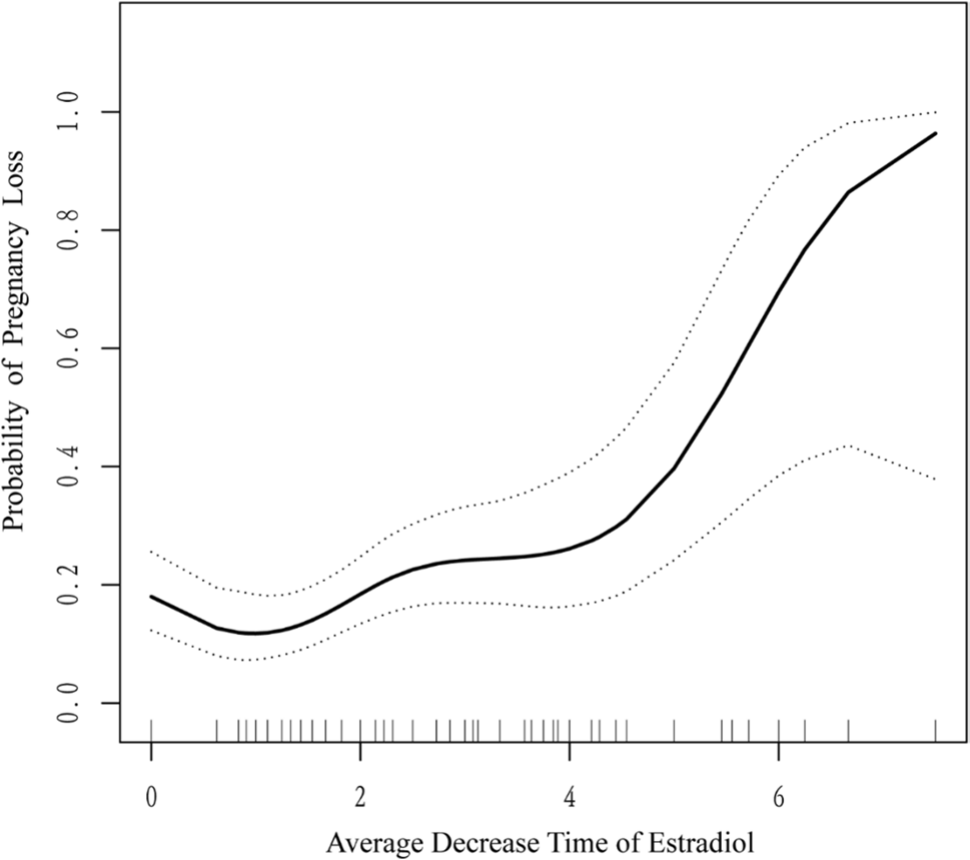
The smooth fitting curve of the average decreased times of E2 and early miscarriage

- Step 1: Chi-square test (categorical variable), Student t-test (normal distribution), or Mann-Whitney U test (skewed distribution) were used to calculate the differences in baseline characteristics of patients with different pregnancy outcomes (Table 1). Serum E2, progesterone, and hCG were measured for the first time.
- Step 2: Multivariate analysis was performed using binary logistic regression. Two models were constructed in Table 2. In Model 1, maternal age and number of previous miscarriages were adjusted. In Model 2, maternal age, number of previous miscarriages, basic hCG, basic E2, and basic progesterone were adjusted. The first measured serum levels of E2, progesterone, and hCG were used as basal serum indicators. To verify the result of ADTE as a continuous variable, we converted the ADTE into a categorical variable by 3-quantile and calculated the *P*-value of the trend.
- Step 3: The nonlinear relationship between ADTE and pregnancy outcomes before 12 weeks of gestation was studied using binary logistic regression and smoothed curve fitting methods, as shown in Table 3 and Fig. 2. When a nonlinear relationship was detected, we used two-piecewise binary logistic regression and binary logistic regression to fit the association and selected the best-fitting model based on *P* for the log-likelihood ratio test. Then, we employed a recursive algorithm to calculate the inflection point and constructed two-stage linear regression models on both sides of the inflection point.
- Step 4: We did a sensitivity analysis to discuss the robustness of the above findings in different subgroups. A subgroup analysis was performed on the number of previous miscarriages and whether E2 decreased continuously (three or more consecutive decreases).

**Table 1:** Baseline characteristics of all study patients

Exposure	Ongoing pregnancy *n* = 359	Early miscarriage *n* = 104	*P*-value
Age (years old)	29.96 ± 3.81	30.12 ± 3.05	0.700
BMI (kg/m^2^)	22.16 ± 2.99	22.42 ± 2.84	0.438
Age at menarche (years old)	13.41 ± 1.11	13.40 ± 1.31	0.949
Regularity of menstruation			0.359
Regular variation	316 (88.0)	88 (84.6)	
Irregular	43 (12.0)	16 (15.4)	
Previous live birth			0.322
0	301 (83.8)	86 (82.7)	
1	54 (15.0)	16 (15.4)	
2	4 (1.1)	2 (2.0)	
Previous miscarriage			0.492
1	130 (36.2)	35 (33.7)	
2	150 (41.8)	50 (48.1)	
≥ 3	79 (22.0)	19 (18.3)	
Type of miscarriage			0.744
Primary	295 (82.2)	84 (80.8)	
Secondary	64 (17.8)	20 (19.2)	
Basic hCG	2114.0 (457.5-13404.0)	1180.0 (238.5-6371.0)	0.027*
Basic progesterone	29.28 ± 12.18	26.99 ± 14.83	0.112
Basic E2	443.77 ± 347.12	401.54 ± 342.29	0.291
ADTE	1.82 ± 1.47	2.91 ± 2.04	<0.001*
Medicine	309 (86.1)	88 (84.6)	0.708
Disease			0.449
None	335 (94.1)	103 (99.0)	
Autoimmune diseases	6 (1.7)	1 (1.0)	
Endocrine diseases	8 (2.2)	0	
Gynecological diseases	3 (0.8)	0	
Other diseases	4 (1.1)	0	

Data are shown as mean **±** standard deviation, median with interquartile range, or frequency with percentages. *BMI* body mass index, *ADTE* the average decreased times of E2

*Statistically significant difference

**Table 2:** Association of the average decreased times of E2 and early miscarriage

Exposure	Model 1^a^		Mode 2^b^	
	OR (95%CI)	*P*-value	OR (95%CI)	*P*-value
ADTE	1.344 (1.156-1.561)	<0.001	1.346 (1.154-1.571)	<0.001*
ADTE tertile				
Low (0-1.25)	Ref	-	Ref	-
Median (1.25-2.5)	1.278 (0.675-2.417)	0.452	1.249 (0.650-2.339)	0.505
High (2.5-7.5)	2.193 (1.171-4.104)	0.014	2.170 (1.144-4.117)	0.018*
*P* for trend		0.012		0.015*

*ADTE* the average decrease times of E2

*Statistically significant difference

a: Adjust for age, and previous miscarriage

b: Adjust for age, previous miscarriage, basic hCG, basic E2, and basic progesterone

**Table 3:** Threshold effect of the average decreased times of E2 and early miscarriage

Exposure	OR (95%CI)	*P*-value
Fitting model using a standard binary logistic regression model	1.346 (1.154-1.571)	<0.001*
Fitting model using two-piecewise regression model		
Cutoff point	4.90	
<Cutoff point	1.199 (1.002-1.436)	0.047
≥Cutoff point	5.713 (1.255-23.170)	0.024
P for log-likelihood ratio test		0.013

## Results

### Baseline Characteristics

Table 1 shows the baseline characteristics for the 463 included participants. For the three basic indicators of pregnancy, the case group’s basal hCG level was significantly lower than that in the control group, while no important difference was reflected in progesterone and E2. Additionally, the ADTE in patients with EM was notably higher compared with control group. No significant differences between the two groups were observed in age, menarche regularity, previous live birth, number and type of previous miscarriages, history of diseases, and medication history.

### The Relationship between ADTE and Early Pregnancy Outcomes

Logistic regression was used to discuss the effect of average decreased times of E2 on early miscarriage (Table 2). The results showed the ADTE increased the rate of EM demonstrably (OR = 1.344, 95%CI 1.156-1.561), and this conclusion remained after adjusting for basal hCG, progesterone, and E2 (OR = 1.346, 95%CI 1.154-1.571), indicating the results were stable. After grouping the ADTE three quantiles, the subjects had a statistically significant increase (*P* = 0.012, 0.015, respectively) in the rate of EM with the increased ADTE in the two models.

### The Threshold Effect of ADTE on EM

ADTE has a nonlinear relationship with EM by logistic regression smooth-fitting curves, as shown in Table 3. And we further identified the threshold as 4.9. For each additional unit of ADTE, the multiplicity of EM increased by 0.199 for ADTE < 4.9 (OR = 1.199 95%CI 1.002-1.436) and 4.731 for ADTE > 4.9 (OR = 5.713 95%CI 1.255-23.170). The data stressed ADTE with a threshold effect—its influence on EM changed dramatically when ADTE exceeded 4.9. There were meaningful differences between the ADTE < 4.9 model and ADTE > 4.9 model (*P* for log-likelihood test = 0.013), the former impacted EM slightly, yet the latter notably.

### Subgroups Analysis

ADTE’s influence on EM was similar among subjects with one, two, and three or more previous miscarriages (OR = 1.447, 1.265, 1.422, respectively) in Table 4. Moreover, we divided patients into two groups based on whether a consecutive drop of E2 existed, and found the effect of ADTE on EM in the continuously decreasing group was lower compared to the non-continuous decreasing group.

**Table 4:** Subgroup analysis of previous miscarriage and continuous decrease in E2

Subgroup	Number	Miscarriage, n (%)	OR (95%CI)
Previous pregnancy loss			
1	165	21.21	1.447 (1.090-1.920)
2	200	20.00	1.265 (1.016-1.575)
≥ 3	98	19.38	1.422 (0.972-2.079)
Continuous decrease			
Yes	95	27.36	1.300 (1.081-1.642)
No	368	21.19	2.215 (1.350-3.634)

Continuous decrease refers to a sequential decrease in E2 levels on 3 consecutive tests.

## Discussion

EM is one of the most common pregnancy diseases with potentially devastating effects on patients. However, valid indicators to measure the risk of EM are still lacking. Our study aimed to investigate the relationship between ADTE and EM (before 12 weeks of gestation) in women with a history of miscarriage. We found that ADTE was negatively associated with EM with a threshold effect. When ADTE > 4.9, the EM rate increased 4.713-fold for each unit increase. Our findings showed some value in the diagnosis and treatment of early miscarriage in patients with previous miscarriages.

E2 is very important for a healthy pregnancy, such as making the endometrium prepared for embryo implantation, regulating the maternal-fetal immune system, and facilitating the fertilized egg to the uterine cavity [18–21]. For a long time, most scholars believed that before 6 weeks of pregnancy in women, the production of E2 was mainly secreted by the corpus luteum of the ovary. As pregnancy progresses, due to the gradual formation of the fetal-placental system, E2 slowly transforms into being secreted by the fetal placenta [20, 22, 23]. The decrease of E2 is associated with adverse pregnancy outcomes. Furthermore, many studies found that the E2 levels of normal pregnant women, threatened abortion pregnant women and abortion pregnant women decreased in early pregnancy, suggesting that the lower the serum E2 level, the worse the pregnancy outcome [5, 24]. Serum E2 levels were significantly lower in women with early threatened abortion and ectopic pregnancy than in women with normal pregnancies [1, 24]. And E2 levels can be used alone or in combination with hCG to predict pregnancy outcomes [24–26]. However, although these studies suggest that serum E2 is associated with pregnancy outcome, they focus on serum E2 levels at a specific moment or period may be selection biased. Our study, differently, discussed the association between ADTE within 12 weeks of gestation and miscarriage with a more rigorous trial design. The results showed that ADTE was positively associated with EM. Further subgroup analysis reflected frequent decreases (≥ 2.5) in serum E2 levels had a significantly greater effect on EM than occasional decreases (0-2.49), indicating a continuous effect of ADTE on pregnancy.

There was a threshold effect between ADTE and EM risk, with a sharp increase in EM after ADTE > 4.9. As illustrated in Fig. 2, the effect of ADTE on EM became more significant as the previous miscarriage number increased—a relatively flat risk of miscarriage before 4 times and a steep increase after 4 times. We then calculated the threshold value was 4.9 times, and the rate of EM increased 4.713-fold for each 1-unit ADTE increase when ADTE > 4.9 times.

In subgroup analysis, ADTE had similar effects in patients with different numbers of miscarriages (OR = 1.447, 1.265, 1.422). ADTE was a risk factor in patients with different numbers of miscarriages, suggesting the robustness of E2. Surprisingly, ADTE in patients with 1-2 miscarriages had a greater effect on EM compared to patients with 3 miscarriages. This may be an accident event caused by the small sample size of patients with 3 miscarriages. Meanwhile, discontinuous decreases in E2 had a greater effect on EM than in patients with continuous decreases in this study, though a continuous decrease in E2 levels is generally considered to be a red flag. There are two possible reasons for this: first, the sample size of the continuous decrease group was much smaller than that of discontinuously decreasing patients, so a chance of error may exist. Second, the baseline E2 levels, and the magnitude of each decrease, were not addressed herein and may also be subject to chance. In conclusion, ADTE did have a significant effect on pregnancy outcomes, and this trend was observed in all subgroups.

We determined a new index to evaluate EM and guide clinical practice. ADTE use allows more direct observation of the relationship between various E2 decrease times and the risk of miscarriage, providing important information on pregnancy outcomes for patients and clinicians. It was also the first time that EM was predicted in patients with a history of miscarriage. Notably, the major strength of this study was the large sample size, including more than 463 pregnancies, ensuring that different pregnancy outcomes were adequately analyzed. However, there were several limitations to our study. First, the population of this study was limited to women with a history of miscarriage, so extrapolative validation tests for normal women were necessary. Second, due to diagnostic difficulties, missing or uncollected data, and diseases that may cause decreased E2 (e.g. luteal insufficiency, ovarian dysfunction, etc.) were neither considered nor adjusted for in the analysis. Furthermore, we did not exclude the patients with vitamin D deficiency. A meta-analysis showed that vitamin D deficiency and insufficiency are associated with miscarriage [27]. And several studies indicated that vitamin D is associated with E2 level [28, 29]. Further well-designed studies are needed to validate our findings. Finally, we did not study the decreased magnitude of E2 levels, some decreases may be a chance, which impacts our study results.

## Conclusion

This study is the first to focus on the relationship between times of E2 decrease and the risk of EM in women with a history of miscarriage. Results suggested ADTE was negatively associated with EM in women with previous miscarriages with a threshold effect. Although our findings suggest that ADTE has the potential as a predictor, caution is necessary due to study limitations. Further prospective research is imperative to validate and refine these preliminary observations for improved clinical relevance.

## Data Availability

The datasets used and/or analyzed during the current study are available from the corresponding author on reasonable request.
